# Introducing Computerized Technology to Nurses: A Model Based on Cognitive Instrumental and Social Influence Processes

**DOI:** 10.3390/healthcare11121788

**Published:** 2023-06-17

**Authors:** Becky Tsarfati, Daniela Cojocaru

**Affiliations:** Department of Sociology and Social Work, “Alexandru Ioan Cuza” University of Iasi, 700506 Iasi, Romania

**Keywords:** nursing, Technology Acceptance Model (TAM), Electronic Medical Systems (EMS)

## Abstract

The use of computerized technologies as an integral part of nursing has become a reality in the health care system. Studies present different approaches that range from accepting technology as a health promoter to an approach that opposes computerization. This study, which examined social and instrumental processes that influence nurses’ attitudes toward computer technology, will present a model for the optimal assimilation of computer technology in the nurses’ work environment. The study, which included 224 participants, was designed as a mixed method and included questionnaires and semi-structured interviews of participants. The data were analyzed to understand the factors that influenced nurses’ attitudes toward the use of computer technology. The research findings show that the more clearly nurses understand the positive impact of using technology on the quality of care, the more positive their response to changes in registration and reporting methods. It is not surprising that the research findings found that cognitive instrumental processes and social influence processes have a positive effect on the perceived usefulness of using computer technologies. The unusual finding was the fact that cognitive instrumental processes were the main factor influencing the assimilation of computer technology even though nursing is a social profession.

## 1. Introduction

The introduction of advanced computer technologies into health services necessitates a change not only in the structure of the organization and job definitions within the organization, but also at the level of the individual, reflected in the need for individuals in the organization to change day-to-day processes.

The use of computerized technologies as an integral part of nursing has become a reality in the health care system. Studies present different approaches that range from accepting technology as a health promoter to an approach that opposes computerization, where it is perceived as a system that takes nurses away from the patient. It was expected to think that young nurses with a lot of experience in computer use would be more positive towards introducing technology into their work environment. Computerized systems in the work of the nurse introduced technological challenges into the nursing profession. Challenges that require adaptation to new working methods, expansion of knowledge and skills in the field of technology, and integration of all this in quality care, create professional and personal frustrations [1,2]. The accelerated development of technologies in the field of science have led nurses to the need for adjustment and change in the therapeutic care approach and improvement of their abilities to use computerized technologies. There is no debate at all among nursing professionals that the main goal of introducing computerized technology is to improve the quality of care for patients [3].

The rapid development of information technologies and their introduction into the health systems at all levels of service providers has affected not only working nurses but also nursing students and teams that educate in the teaching frameworks [4]. The question that arises is not whether computerized technology should be used, but what is the best way to integrate computerized technology to achieve the most positive impact on nursing practice.

Successful application of computerized technology is a product of the attitudes of the users of the technology; therefore, it is very important to examine the attitudes of nurses and understand the factors that influence these attitudes. The results of studies on this topic will allow the construction of implementation and adaptation plans that allow the introduction of technological changes into the nurses’ work environment, both in nursing educational settings and in nurse enrichment settings [5].

Nursing is a profession with a very strong social affinity and as such nursing has a social obligation to provide health services based on growing knowledge and professional skills. The nursing profession focuses on maintaining the health of the population, disease prevention, and holistic treatment while maintaining an ethical code. Defining the limits of nursing practice requires nurses to be up to date on the development and progress taking place in health services that enable quality treatment options. The academic study framework prepares nursing students to become professionals with independent thinking abilities and decision-making abilities based on extensive integrative knowledge; therefore, nurses are obliged to be up to date on technological developments that enable the implementation of these goals [6,7].

Studies [8,9,10,11], have found that the fact that nurses were not partners in creating the computerized system led to dissatisfaction with its use since the computerized system did not meet the professional needs of the nurses. Nurses who are the end users of computerized registration and reporting systems were not actively involved in building the contents of the system and therefore difficulties in use were created and, in some cases, it was found that there was resistance to the use of the computerized method [12].

The main question examined in the study was “What factors are involved in nurses’ acceptance to use new technological systems?”

The main contribution of this research is manifested in several levels:

- Understanding processes that led to the positive perception towards computer technology among nurses.
- Establishing the assumption that the quality of care is an integral part of the essence of the nurse’s role.
- Presenting a model that combines instrumental and social influence processes to ensure nurses’ readiness to accept the technology and allow adequate support to maintain technological skills for nurses.

## 2. Literature Review

Technological progress and changes in the health care system oblige nurses, as part of the set of caregivers, to adjust their roles [13,14,15]. Nurses are required to stay up to date on working practices in a computerized environment and improve their access to digital information [16]. Since the entry of computerized technologies into the professional lives of nurses, many studies have been conducted to examine the responsiveness, limitations, or difficulties created because of the use of computerized methods for documentation and reporting. In research on health services conducted in Turkey, it was found that the use of computerized technologies in the health system was not at the desired level among nurses because the technologies were not compatible with the needs of nursing practice. The conclusions of this study indicated the need to make computerized technology accessible to nurses by involving the nursing staff in building the relevant software [17].

Studies that examined the impact of computerized technologies on the work of nurses showed that the common perception is that the introduction of computerized technology will lead to a reduction in care errors and improve the quality of care provided [2,15]. McGonigle et al. [18], found that nurses’ use of computerized technologies allowed them to make updated and improved clinical care decisions in patient care. The introduction of technology that changes existing working habits is likely to bring difficulties in its implementation, as shown in a study conducted in two Australian hospitals. The study presented the main difficulties of nurses regarding the use of Electronic Medical Systems (EMS) and identified technical access barriers, lack of knowledge of technological systems, and a conflict in the nurses’ professional identity as part of the difficulties faced by the nursing staff. Research participants stated that using EMS threatens their identity and professional image, as they were educated to make independent decisions and think critically [8].

Nurses make up a high percentage of those employed in the healthcare system and at the same time are the end users of technological innovations introduced into the field of healthcare provision; therefore, it is very important to understand barriers or factors that promote behavioral change that result in a maximum ability to work with computerized technology [19]. Nursing is a profession with a very strong social affinity, and as such, it has a social obligation to provide health services based on knowledge and increasing professional skills. At the same time, nursing is a profession of teamwork, and the role of the nurse is built based on learned behaviors, so it is extremely important to understand the interrelationships between nursing and society and the definition of the role studied as part of the core studies. We believe that understanding the processes that lead nurses to positively integrate technologies will help introduce those technologies into the healthcare system and lead to improved quality of care.

In nursing, we find the need to balance the duality of self and society as a basis for the development of professional identity. As people with extensive professional knowledge, nurses have a conscious self-concept of themselves in the nursing profession. However, as part of the nursing society, each nurse must act in the workplace by society’s norms [20]. The study examined the degree of influence that cognitive instrumental processes and social processes have in the application of computer technology through the Technology Acceptance Model [21,22].

### 2.1. Technology Acceptance Model

The TAM [21,22] is a model that focuses on the intent of the end users and predicts the response of these users to a required change. This is a very common model for forecasting in healthcare systems. The Technology Acceptance Model (TAM) was used in this study to understand the factors influencing the acceptance of new electronic technologies among nurses.

The TAM assumes that the two basic specific components—perceived usefulness and perceived ease of use—are factors that influence the acceptance of change in general and the decision to use computerized technology by nurses according to this study. According to Davis [21], for these two components there is a primary relevance to technology acceptance. Perceived usefulness is defined as the degree to which people believe that using a certain information system or a certain technology will improve their work performance. Studies investigating the adoption of new technology have shown that perceived usefulness was a decisive factor in the users’ decision in this context [23]. Perceived ease of use is the individual’s expectation that the information system or technology will be easy to use for them. This is one of the factors that predict the intention to use computer technology [24]. It is important to note that this decision also depends on the influence of external factors such as professional seniority, norms, and procedures in the work environment [25].

To overcome the limitations of the initial model, Venkatesh and Davis [26], expanded the original model, calling it TAM2. The expanded model incorporates two additional groups of factors that influence the adoption or rejection of the use of computer technology. The first group relates to social influence and includes the elements of subjective norms, experience, desires, and image. The second group, known as a cognitive instrumental influence, includes the elements of role relevance, quality of output, and demonstration of the result, as shown in Table 1.

The study examined the degree of influence of these two groups of components with the hypothesis that social components will have a greater impact on behavioral change among nurses.

#### 2.1.1. Social Influence Processes

Social processes affect the individual’s behavior at different levels and include subjective norms, image, volunteering, and experience.

A subjective norm is defined as the individual’s perception of people who are important to them, for example, whether to adopt a change in behavior about computer use [27]. Venkatesh and Davis [26] linked subjective norms and voluntarism and stated that people’s behavior is not always voluntary, but follows the influence of significant others. Even if people do not want to behave in a certain way, they will change their behavior if they believe that changing behavior is important to significant others. Venkatesh and Davis [26] made a connection between subjective norms and perceived usefulness. This is based on the definition of the concept of internalization and adaptation, which implies the belief of individuals in the importance of behavior concerning significant others in the social sphere, the system, or the organization to which they belong.

The second social effect—image—is defined as the people’s belief that using technology as an expression of a change in their behavior will improve their status in the organization where they work. This perception links subjective norms and images. People who believe that using a computerized system will advance their position in the organization will have more willpower to perform the task, improve productivity, and thus increase the perceived benefit [28].

The third social process—voluntariness—is defined as a concept that links a subjective norm to intentional behavior, that is, adopting a certain behavior even when the individual does not believe in it. When the use of the system was not mandatory, there was no direct relationship between subjective norms and behavioral intention. However, when the use of the system was mandatory, a clear direct relationship was found between subjective norms and behavioral intention. That is, when the use of the system was mandatory, the behavior change was related to the perception of significant others for them. Whereas, when the change was adopted voluntarily, the intention to change behavior was determined according to the attitudes of everyone without giving importance to the perception of others [28].

The last social process—experience—is a linking factor between subjective norms, perceived usefulness, and behavioral intention. Venkatesh and Davis [26] suggested that the more experience people have using a system, the less they will be influenced by significant others with respect to behavioral intention, perceived usefulness, and subjective norms.

#### 2.1.2. Cognitive Instrumental Influence Processes

Venkatesh and Davis [26] claim that people decide to behave in a certain way, based on the understanding that instrumental behaviors are related to goals at a higher level than a simplistic demonstration of behavior.

The first element, job relevance, is defined as the individual’s perception of the applicability of the technology and the existing match between the job requirements and the technological capabilities. Job relevance has a positive effect on perceived usefulness.

The output quality component is defined as the individual’s perception of the quality of the system in performing a specific task. In contrast to job relevance, output quality implies that, given an individual a choice between two systems, a person will always choose the system with the highest quality output. Therefore, quality output has a positive effect on perceived usefulness.

The last component is result demonstrability, and is defined as a situation where people have a positive attitude towards the usefulness of the system when they can distinguish between use and a positive result. Result demonstrability is like a situation where people perceive that the system will be more useful only when they can easily understand the effect of using the technology [26]. According to Venkatesh and Davis [26], their TAM2 model shows that cognitive instrumental processes will have a positive effect on perceived usefulness and, consequently, will have a positive effect on people’s intention to use the system.

Holden and Karsh [29] present in their review that the TAM in all its versions was found to be the most suitable model for predicting the adoption of information technologies by healthcare providers. The model has a high ability in predicting the responsiveness of healthcare teams to adopting new technologies. A strong positive relationship was also found between attitude and behavioral intention and between behavioral intention and use. Furthermore, the findings showed a weak relationship between the perceived ease of use of the approach and the perceived ease of use of the behavioral intention. The researchers suggested that these findings were due to the participants’ lack of experience using the technology.

Perceived usefulness consistently predicted the use and adoption of new technology among different health professionals, and perceived ease of use correlated with perceived usefulness. At the same time, no consistent results were found between the perceived ease of use and the adoption of information technologies, due to inconsistency and inability to use technology, or differences in work [29]. Using the Technology Acceptance Model 2 questionnaire allows us to present the influence of the instrumental cognitive processes and the influence of the social processes in a clear and defined way.

### 2.2. Electronic Medical Systems

Digital technologies refer to electronic information sources and the Internet whose purpose is to optimize access to medical and nursing information for the use of health consumers and health system staff. Digital health includes many categories such as mobile health (mHealth), health information technology (IT), telehealth, and telemedicine. Digital health technologies have a wide variety of uses and are implemented on different platforms to serve as tools for providing fast and high-quality comprehensive care [30].

There are many benefits to using EMS as opposed to nurses’ use of manual registration and reporting [31]. The prevailing perception is that the quality-of-care increases as nurses have easy, fast, and accurate access to their patients, and this is made possible by using EMS in the daily work of the nurses. In addition, it is possible to reduce the chance of mistakes in nursing practice following the use of online sources that allow clear writing of care instructions. The degree of satisfaction with the ability and ease of use and understanding of the purpose of using computerized technologies in the work of the nurse can explain the related behavior of the nurses about the use of computerized information systems [23].

Among the variety of digital technological options available, the current study focused on computerized registration and reporting as part of Electronic Medical Systems (EMS). The choice of this technology was due to familiarity with the practice of nursing and an understanding of the centrality and importance of registration and reporting by nurses in their daily work.

## 3. Materials and Methods

A non-experimental mixed method study was used in this study. The study was conducted among nurses working in hospitals in various departments and nurses working in community clinics in Israel. The aim of the study was to understand the degree of influence of cognitive processes and social processes due to the introduction of electronic registration and reporting to the nurse’s work. The first phase of the research included sending questionnaires via email, and was conducted from December 2020–February 2021. The second phase of the research included semi-structured interviews and was conducted from March 2021–May 2021. Health research is often based on a quantitative method and has several advantages. The main advantage of quantitative methods is their usefulness in producing factual and reliable outcome data. The statistical methods associated with quantitative research can find ways to maximize dependent variables based on independent ones [32]. The addition of the qualitative approach in this study made it possible to investigate, explain, and describe behaviors within the context in which they occur, with the aim of obtaining objective results that can be generalized to larger populations.

**Figure 1:**
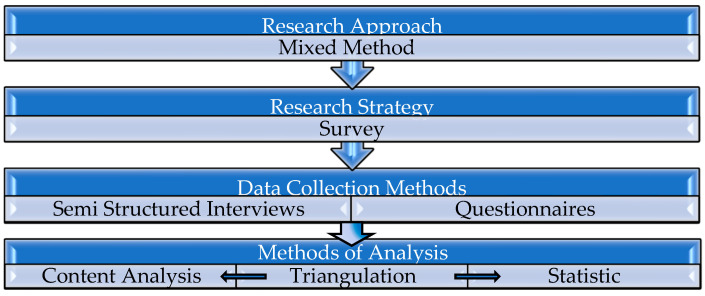
Research Methodology Stages.

The quantitative part is intended to present the processes that influenced the acceptance of EMS in the work of the nurses and the qualitative part is intended to explain the processes that were perceived by the nurses as significant processes in the acceptance of computerized technology from the personal experience of the interviewees, as explained in Figure 1.

The article aims to present the main factors for accepting a behavioral change among nurses with the understanding that the quantitative findings will be able to reflect the reasons for which nurses are ready to make a fundamental change in their work. The purpose of the research is to create structured processes for introducing computerized technology or any behavioral change in an organized and systematic manner. The qualitative part contributed to the creation of a model according to which it is possible to make a change in work processes with the help of interviews that allowed the participants to present their personal experiences during the introduction of a computerized technological change.

The study was conducted only after receiving approval from the ethics committee on behalf of “Alexandru Ioan Cuza” from the University of Iasi.

### 3.1. Data Collection

This article is based on an analysis of information collected through questionnaires that examined the acceptance of the change in registration and reporting in nursing practice from manual registration to electronic registration.

The quantitative part of the study was conducted using questionnaires examining the use of computer technology by extending the Technology Acceptance Model questionnaire [26]. The questionnaire also included demographic information such as age, gender, education, years of experience, and workplace (hospital or community health services).

The questionnaire was collected in a survey research strategy to enable rapid data collection for a large population group of nurses using e-mail. Data were collected by using a convenience sample method.

The qualitative data were collected through semi-structured interviews (Appendix A) after each participant expressed consent to the interview, and received an explanation about the research and the interview. Since the study was conducted during the period of COVID-19, the interviews were conducted by telephone separately for each participant. They were recorded and transcribed, then the recordings were deleted to preserve the privacy of the participants. To create a uniform framework, the interview questions were formulated according to a general interview guide [33]. The interview included questions about personal details and professional background, such as age, gender, workplace, and years of experience. In addition, the interview included open-ended questions to allow the interviewees to present their personal experiences regarding coping with a change in their professional practice. Each interview lasted 45–90 min, depending on the amount of time each interviewee felt comfortable answering the questions.

### 3.2. Participants

The quantitative phase consisted of a questionnaire sent via the Internet to Israeli nurses using the survey method to allow a wide application within a short period and to allow anonymity of the participants.

An adequacy test was performed using the G-Power software for the regression model in the quantitative phase of the study based on the data: Effect size—015, a—0.05, Power—0.80, and 7 predictors. A total sample size of 103 was found. The actual study included 214 participants, as shown in Table 2, with 88% females, between the ages of 24 and 65 years. The nurses were 42 years old on average (*SD* = 9.51) and have been working in the field for an average of about 15 years (range = 1–35 years, *SD* = 9.92). Most had full-time employment (86%) and worked in a hospital (84%), in diverse departments. About two-thirds of the departments were general ones (e.g., surgery, children, internal) and one-third were departments that required professional studies (e.g., intensive care, operating room, psychiatry). Community employment (*N* = 35) was in a general health clinic (*N* = 22, 62.9%), or a professional clinic (*N* = 13, 37.1%).

As shown in Table 3, 10 participants in the qualitative phase were interviewed in the second phase of the study. This phase consisted of semi-structured personal interviews of nurses randomly and voluntarily selected in community centers and hospital wards. This sample included nurses from hospitals and community nurses and matched the percentages of participants in the quantitative part in the distribution to the community and the hospitals as detailed below. The average age of all participants was 44.3; 70% were women aged 29–65, and the remaining 30% were men, aged 34–61. The average seniority of the participants was 19.5 years; 20.7 years for the female participants, and 16.6 years for the male participants. A total of 70% of the participants worked in a hospital: 67% of them worked in general wards and the rest worked in special care units. A total of 35% of the participants worked in community clinics.

### 3.3. Instruments

The data for the quantitative part of the study was collected using the TAM2 Measurement Scales questionnaire [26]. The questionnaire examined the nurses’ responsiveness to the use of computer technology on three levels: 1. “Perceived usefulness”: The individuals’ belief that using a particular information system or technology would improve the job performance. 2. “Perceived Ease of Use”: The expectation that the information system or technology is easy to use. 3. “Attitude toward using”: The behavioral intention to use, which can be understood as technology acceptance [29].

The questionnaire is a 26-items questionnaire measuring the perceived usefulness of technology usage, in terms of social influence and cognitive instrumental processes. Its nine subscales are: intention to use, perceived usefulness, perceived ease of use, job relevance, output quality, result demonstrability, subjective norm, voluntariness, and image. Items are rated on a 7-point agreement scale, ranging from 1–strongly disagree to 7–strongly agree (Appendix A). The instrument was found by Venkatesh and Davis [26] to have high internal consistencies (Cronbach α = 0.80 to 0.97). In a longitudinal study of 156 workers in four organizations the measure was found to have high construct validity, and the TAM2 model was supported for the four organizations at three time points [26]. Venkatesh and Davis [26] concluded that “both social influence processes (subjective norm, voluntariness, and image) and cognitive instrumental processes (job relevance, output quality, result demonstrability, and perceived ease of use) significantly influenced user acceptance” (p. 186). Acceptable to high internal consistencies were found in the current study: Perceived usefulness: Cronbach α = 0.92, Perceived ease of use: Cronbach α = 0.76, Job relevance: r = 0.71 (*p* < 0.001), Output quality: r = 0.61 (*p* < 0.001), Result demonstrability: Cronbach α = 0.84 (excluding item 26), Subjective norm: r = 0.74 (*p* < 0.001), and Image: Cronbach α = 0.69.

## 4. Results

### 4.1. Cognitive Instrumental and Social Influence Processes and the Perceived Usefulness of Technology Use

The research question on processes affecting the acceptance of computerized technology by the nurses was examined according to the TAM2 model, and focused on the relationships between cognitive instrumental processes, social influence processes, and the perceived usefulness of the use of technology. This was tested with a hierarchical multiple regression of the perceived usefulness of technology use, with the domains of cognitive instrumental processes and social influence processes. The regression model was calculated while controlling seniority in nursing and gender, as related to the main variables of the study. They were entered in stage 1 of the regression model, while the fields of instrumental and social influence processes were entered in stage 2, as shown in Table 4.

The regression model was found to be significant, with 47% of the variance in the perceived usefulness of the use of technology explained in it. The domains of cognitive instrumental processes and social influence processes added 34% to the explained variance in perceived usefulness, beyond seniority in nursing and gender. Significant and positive relationships were found between perceived usefulness and seniority in nursing, perceived ease of use, relevance to work, and quality of output, so seniority in nursing, perceived ease of use, perceived work relevance, and higher quality of output are related to the greater perceived usefulness of the use of technology. Interestingly, all significant relationships represent cognitive instrumental processes, whereas social influence processes were unrelated to perceived usefulness.

### 4.2. Acceptance of Technology

As shown in Table 5, acceptance of technology was found to be rather high among the nurses, ranging between 4.45 and 5.98, for the various items (SDs = 1.19 to 1.77, score range 1–7). Results show that the highest mean scores were found for ‘job relevance’, ranging between *M* = 5.86 and *M* = 5.98. Next were the mean scores for ‘result demonstrability’, ranging between *M* = 5.61 and *M* = 5.76; ‘perceived usefulness’, ranging between *M* = 5.32 and *M* = 5.64; ‘output quality’, ranging between *M* = 5.25 and *M* = 5.54; ‘perceived ease of use’, ranging between *M* = 4.92 and *M* = 5.56; and ‘subjective norm’, ranging between *M* = 5.15 and *M* = 5.21. The lowest was the mean score for ‘image’, ranging between *M* = 4.45 and *M* = 4.74. That is, about 60% to 65% of the nurses agreed that the use of technology was relevant and important to their work. Others had a neutral opinion on this matter, and almost none disagreed with it. Regarding ‘perceived usefulness’, about 50% of the nurses claimed that the use of technology was effective and beneficial, while most others had a neutral opinion on this matter. Quite similarly, about 50% of the nurses agreed that they received high-quality output from the system, while most others were neutral about it. Concerning ‘perceived ease of use’, close to 50% of the nurses, on average, agreed that using the system was easy for them, and only a few resisted it. ‘Subjective norm’ was somewhat lower, with about 42% of the nurses, on average, agreeing that important others supported the use of technology, and about 54% expressing a neutral opinion. Finally, only about 32% of the nurses thought that using the system was related to a high status or a positive image. Most, about 60% of them, had a neutral opinion, and about 10% expressed disagreement. Among the different subscales, two were not suitable for the study: volunteering and intention to use. Volunteering was not appropriate as the use of EMS is mandatory. Indeed, Venkatesh and Davis [26] used this scale to verify the distinction between organizations where the use of EMS was mandatory and those where its use was voluntary. Intention to use was also inappropriate, as EMS among study participants had already been introduced and nurses were required to use it.

The main finding obtained from the data showed that cognitive instrumental processes were the main reason for the perceived benefit of using computer technology. Significant and positive relationships were found between perceived usefulness and seniority in nursing, perceived ease of use, relevance to work, and quality of output. That is, the higher the seniority in nursing, the more perceived ease of use, the more perceived job relevance, and the higher quality of output, were related to the more perceived usefulness of technology use [1].

This finding is also supported by the interview data which indicated that nurses with professional seniority accepted the computerized technology as having a positive effect on the care provided. Thus, despite difficulties in adapting and sometimes a lack of technological knowledge, as well as a lack of relevant professional support, nurses still expressed satisfaction with the changes in the registration and reporting method, as shown in Table 6 and Table 7.

The nurses stated during the interview that quick access to extensive information resulted in efficient treatment, and enabled quality treatment while relying on receiving data in a short period. The nurses recognized the use of computerized technology for reporting as a substantial advantage and an essential element for improving nursing practice. Registration and reporting in nursing practice are significant and form the basis for providing quality care. Making accurate, clear, and understandable reports allows for continuous, quality, and appropriate care for each patient, as expected of each nurse. The introduction of electronic registration to the practice of nursing made it possible to prevent errors that appeared in manual registration due to difficulty in clearly understanding the text. In addition, the interviewees stated that the cooperation with computer teams enabled the assimilation of the technology easily and positively despite the adjustment difficulties had by some of the team members.

Regarding barriers to the use of computerized technology, the interviewees stated that a lack of knowledge and inappropriate implementation methods led them to have trouble in implementing as required and as a result sometimes a delay in providing treatment. These factors led to feelings of frustration in some of the interviewees.

## 5. Discussion

The rapid development in the life sciences has resulted in specialization in the field of health and has created a working environment for sharing health information across both institutional and global boundaries. New and advanced innovations at the technological level were introduced into the health systems with a great focus on the technical aspects of system planning and less focus on the human resources that must operate these systems [25]. In addition to the quantitative findings, the qualitative findings provided insight into the impact of the TAM components, such as work relevance, output quality, ability to demonstrate the result, perceived usefulness, perceived ease of use, subjective norm, and experience. These qualitative findings explained the interviewees’ feelings about barriers and factors that promoted their decision to use computer technology. Moreover, they illuminated how nurses faced difficulties and identity dilemmas, arising from computer technology.

Weeger and Gewald [34] claimed that nurses expressed fear and anxiety that the introduction of computers to work would lead to distance from patients, allocate less treatment time, and may even lead to the loss of their job. Registration and reporting are among the key functions in the practice of nursing. Accurate, clear, and understandable registration and reporting allow for continuous, high-quality, and appropriate care for each patient, as expected from each nurse. Until the introduction of electronic registration for the practice of nursing, nursing and medical instructions were recorded manually. This, at times, led to errors due to difficulty in clearly understanding the text. Therefore, computerized registration is seen by nurses as improving the quality of care, preventing mistakes, and technologically advancing them to other professions in the health field.

Findings show that cognitive instrumental processes have a major impact on the acceptance of technology and professional identity rather than social influence processes.

Studies that focus on researching behavior change using the TAM [5,35,36] present the change as involving perceived usefulness and ease of use for understanding the change in behavior. The findings of the present study are like the findings presented in these studies. In addition, the current study found that years of seniority is a decisive factor in accepting technological change among nurses; despite difficulties in adapting and lack of knowledge on the subject, nurses with professional seniority emphasized technical registration and reporting as a tool designed to improve the quality of care.

In this study, the main finding that cognitive instrumental processes had the greatest impact on the use of computer technology presents a different picture than expected according to this theory, which emphasizes the matter of belonging to a group as a main motive in changing behavior. The possible explanation that can be offered for this finding is the nurses’ perception of the patient population as a significant group that must be protected, and therefore there is the perception, mainly among nurses with professional experience, that despite the difficulties of adaptation and differences in the definition of their roles, the behavior change must be accepted positively and adopted to improve the quality of the care provided to the patients.

This information makes it possible to see the nursing profession as having a focus on the population. The main nursing activity is for the preservation of society’s health, as presented in the literature review concerning sociology and nursing.

Due to nursing being a social profession in which professional behavior is related to the behavior of the professional group, it was expected that the research findings would mainly point to social factors as the main influences on nurses’ conduct in technological changes. However, unlike what was expected, there was not a strong correlation between socialization processes and technological changes, but rather the cognitive instrumental effect was significantly greater in the nurses’ considerations for receiving computerized technology. Data analysis showed that socialization processes have less of an effect on the acceptance of technological changes, while cognitive instrumental processes such as perceived usefulness, perceived ease of use, job relevance, output quality, and result demonstrability, were expressed as major factors influencing accepting a technological change. The findings showed that the main factors affecting nurses’ decision to use computer technology were cognitive instrumental processes, such as job relevance, output quality, and result demonstrability.

The first concept was perceived usefulness; the more the nurses perceived that the use of EMS would improve their performance at work, the more positive their attitude toward EMS was. Nurses who perceived the use of computer technology as advancing their professional abilities and increasing quality output changed their behavior. This change is reflected in the positive acceptance of computerized technology in registration and reporting as a positive aid, although sometimes adapting to the system was difficult. The second concept was cognitive instrumental processes, i.e., job relevance, output quality, and result demonstrability.

The findings showed a high level of responsiveness to technology acceptance by the nurses. Sixty to sixty-five percent of the nurses concurred that technology use was important for their work. Evidence for this was manifested by the words of interviewees 1, 3, and 5: “*My experience working with electronic registration is very positive, allows me quick access to the patient’s database. A computerized work environment has created an order for me at work*”, “*Today I have no doubt that nursing is easier for him in electronic registration for me*”, and “*Since the introduction of a computerized sheet, I make the registration and reporting in a clear, and immediate manner. There is access to a lot of data in a very short period, which in my opinion increases the efficiency of the work*”. Fifty to sixty percent of the nurses claimed that they understood very well the meaning of using Electronic Medical Systems (EMS), as well as the efficiency of the system in day-to-day work. Thus, they also understood the importance of healthcare aligned with the use of a computerized system. Interviewees 1, 7, and 10 said: “*The computer optimizes the work, allows accessibility and accuracy*”, “*You can get a lot of information in a short period, the writing is legible and it prevents mistakes in registration. Today I have no doubt that reporting for nurses has become an easy and accurate operation following electronic registration*”, and “*electronic registration allows me quick access to many data and optimizes my work*”.

Similar findings have also been found in previous studies [5,23,36]. A study conducted by Yontz et al. [5] emphasized the importance of nurses’ implementation of EMS. The researchers noted that proper implementation would lead to long-term use of computer technology, entailing an improved nursing practice. A study examining the use of EMS among nurses in the intensive care unit [37] found that responsiveness to the use of technology depended on perceived ease of use and perceived usefulness, as examined in this study.

In addition, strong positive relationships were found between years of experience in the profession and receiving technological change. The most likely explanation for these findings may be the fact that nurses with extensive professional experience come from an educational agenda that nursing is a profession that is always evolving and changing over the years; exposure to these values leads nursing professionals to be flexible with changes that apply in the profession, even in the technological field.

The findings of the qualitative part of this study indicated the same conclusions regarding the use of a computerized system. That is, in the wards where the nurses were allowed to change the software according to their needs at work, there was great responsiveness and a positive perception regarding the use of EMS. Interviewees 2, 4, 5, 7–10 said: “The incompatibility of the software on the computer led the nursing staff to initiate planning for a change in the software and cooperation with the computing unit”, “Acceptable changes were made according to the nature of the work”, “In order not to make a mistake, our department asked the unit the computer to build together a treatment plan for the common and representative condition”, “without cooperation with the computer unit it would have been difficult to get the electronic record”, “we requested the change of the software to adapt to our department”, “the nature of the patients and diseases in the department required a change of the software by the computer unit”, “The team worked more efficiently after a change was made in the reporting software.”

The reason for this may be that, although nursing is a teamwork profession, ultimately the practice of nurses is personal. Dealing with the changing job demands, following the introduction to EMS, was personal, and hence, cognitive considerations had the strongest influence on the nurses’ decision to use EMS.

## 6. Recommendations

The nursing administration should consider establishing committees that would include nurses from various professional levels, to establish policy and chart a path for the beneficial introduction of computerized technology in health institutions.

It is recommended to establish professional development programs for nurses to strengthen their skills and technological abilities.

Health institutions must appoint computer trustees for accompanying and guiding nurses throughout the entire period of the introduction of the technological change.

It is recommended to change the curriculum in nursing education, adding specific courses within the teaching and training framework to create an early familiarity of nursing students with EMS.

We believe that a model for assimilating computerized technology based on instrumental and social influence processes in three stages will lead nursing students and nurses in the health system in an effective way to adopt and integrate technology, and improve the quality of care, which will help in the implementation of the recommendations.

**Figure 2:**
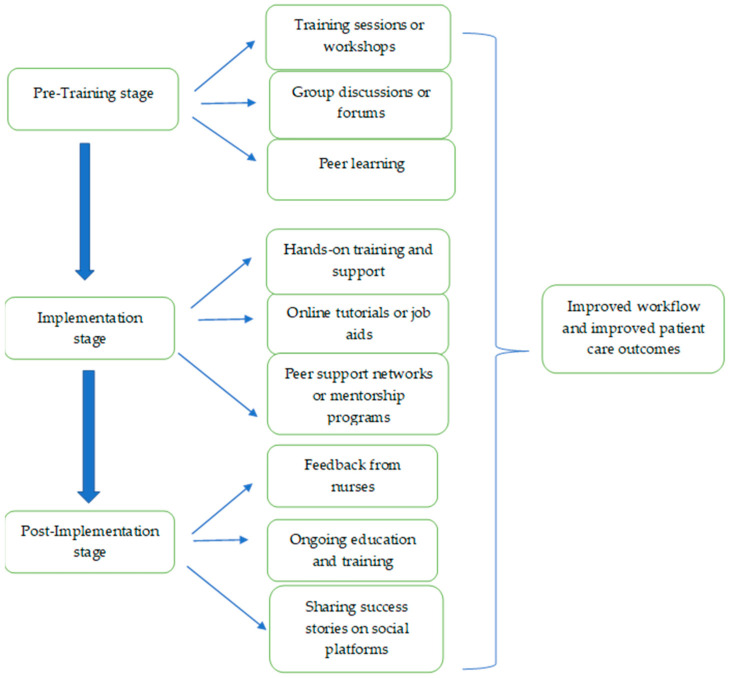
A model that promotes the use of computerized technology for nurses based on cognitive and social processes.

The model shown in Figure 2 allows the introduction of computerized technology to nurses, considering cognitive instrumental and social influence processes. In the pre-training stage, it is necessary to provide cognitive preparation for nurses for the introduction of computerized technology. This preparation will include training and workshops focusing on the theoretical and practical aspects of the technology, its purpose, and its advantages. In terms of social influence processes, it is important to encourage nurses to share their thoughts and concerns about technology in a supportive environment, through group discussions or forums, which allow for the expression of opinions and open dialogue. It is very important to create opportunities to learn from colleagues who have already used similar technologies, with an emphasis on positive social impact.

During the implementation stage, training and practical support throughout the entire period of initial use is of great importance, as is allocating courses and resources, such as online tutorials or job aids, to improve the nurses’ skills in using computer technology. In addition, it is extremely important to give feedback and reinforcement to encourage continued use. In terms of social influence processes, it is important to create a cooperative and inclusive culture while providing encouragement and social support among nurses, including establishing peer support networks or mentoring programs where experienced users can promote and even creating a professional social norm among nurses.

In the last stage (post-implementation), it is extremely important to create assessment processes for the efficiency of the technology from the collected feedback and correct the requirements accordingly. Even at this stage, it is very important to continue training, accompaniment, and support of the nurses to promote learning. In terms of social influence processes, it is very important to continue to foster a supportive culture, providing opportunities to share experiences and best practices with technology.

In conclusion, the model combines instrumental and social cognitive influence processes to ensure the nurses’ readiness to receive the technology, allowing them to support themselves throughout the process, and creating motivation to adopt computerized technology in their daily work.

## 7. Conclusions

The findings from the data analysis show that the social part is not a direct factor influencing the process of accepting computerized technology, but the nurses’ perception of the benefits arising from a technological change in nursing practice. When nurses are required to deal with technological changes facing them independently in front of a computer monitor, nurses are tested on their ability to deal with computerized registration and reporting. Thus, the effect of social influence processes was smaller than that of cognitive instrumental processes. However, when we examined the nurses’ behavior towards the patients, it seemed that a change in professional practice, which resulted in a higher level of care quality, resulted in a positive perception of the change.

The basic assumption that socialization processes have a great impact on receiving change is not reflected in the analysis of questionnaires; this is also the main reason why the study is a combined study and included interviews of nurses, to better understand the processes nurses go through on the way to technological change. The qualitative content analysis provided an in-depth understanding of the adoption of computerized technology among nurses.

Nurses’ decision to use new technology systems was related to their perception that adopting new behaviors functions were relevant to their job, and could improve their performance and advance the quality of care.

In addition, there is great importance in the way in which computerized technology should be made accessible to nurses to improve the personal experience and to promote nursing from a professional point of view. To this end, nurses emphasized that their participation in building the content of the technology and professional accompaniment enabled a quick adaptation and a positive experience.

Another conclusion that can be drawn from the data is the establishment of the understanding among nurses that professional development today depends not only on the conduct of a group of professional colleagues but also personally on individual ability to integrate technological changes. To this end, the nurses expressed their desire to receive relevant support, guidance, and training in the field of computer technology to improve and promote themselves.

## 8. Study Limitations

The study focused on the population of nurses only, and did not include input from the decision-makers regarding the introduction of technological changes.

The study only included a small group of nurses in both the quantitative and qualitative parts, with most of the research participants having professional experience and a relatively older age.

Another limitation is the fact that the study included only one measurement tool; therefore it is difficult to draw comprehensive conclusions. At the same time, this study can form a basis for further studies that will examine the nurses’ motivations for professional behavior in different ways to implement professional changes in the best way.

## Figures and Tables

**Table 1:** TAM2 variables.

Process	Variable
Social influence processes	Subjective norm
	Image
	Voluntariness
	Experience
Cognitive instrumental influence processes	Job relevance
	Output quality
	Result demonstrability

**Table 2:** Quantitative phase participants’ background characteristics (*N* = 214) [1].

Age (years) *M* (*SD*)	42.50 (9.51)
Seniority in nursing (years) *M* (*SD*)	14.87 (9.92)
Gender *N* (%)	
Female	189 (88.3)
Male	25 (11.7)
Current employment *N* (%)	
Full time	184 (86.0)
Part-time	30 (14.0)
Place of work *N* (%)	
Hospital	179 (83.6)
With professional studies (Of *n* = 179)	59 (33.0)
General departments (Of *n* = 179)	120 (67.0)
Community	35 (16.4)

**Table 3:** Interviewee’s background (*N* = 10) [1].

	Name	Gender	Age	Seniority (In Years)	Workplace
1	B.V.	Female	35	9	Community health clinic
2	A.P.	Female	65	43	General hospital dept.
3	H.Z.	Female	38	10	Community health clinic
4	Y.G.	Female	47	22	General hospital dept.
5	A.R.	Male	34	8	General hospital dept.
6	M.T.	Female	56	27	Community health clinic
7	M.L.	Male	43	12	General hospital dept.
8	T.M.	Female	29	2	General hospital dept.
9	A.B.	Male	61	30	Special unit hosp. dept.
10	G.L.	Female	55	32	Special unit hosp. dept.

**Table 4:** Multiple hierarchical regression for the perceived usefulness of the use of technology, with cognitive instrumental and social impact processes (*N* = 198).

	*B*	*SE*	β	*p*	Adj. *R*^2^
Step 1					0.13, *p* < 0.001
Seniority	0.04	0.01	0.36	<0.001	
Gender (male)	−0.32	0.24	−0.09	0.181	
Step 2					0.47, *p* < 0.001
Seniority	0.02	0.01	0.19	<0.001	
Gender (male)	−0.01	0.19	−0.01	0.946	
Perceived ease of use	0.27	0.08	0.24	<0.001	
Job relevance	0.01	0.01	0.19	0.008	
Output quality	0.24	0.06	0.25	<0.001	
Result demonstrability	0.05	0.08	0.05	0.532	
Subjective norm	0.08	0.05	0.09	0.126	
Image	0.03	0.05	0.04	0.480	
*F* (8, 189)					22.47, *p* < 0.001

**Table 5:** Distribution of the items of acceptance of technology (*N* = 205–213).

	Disagree (Scores 1–2) *n* (%)	Neutral (Scores 3–5) *n* (%)	Agree (Scores 6–7) *n* (%)	M (SD)
Perc. usefulness 1	2 (1.0)	93 (44.3)	115 (54.8)	5.64 (1.25)
Perc. usefulness 2	7 (3.3)	105 (50.2)	97 (46.4)	5.32 (1.36)
Perc. usefulness 3	4 (1.9)	90 (43.3)	114 (54.8)	5.50 (1.31)
Perc. usefulness 4	1 (0.5)	94 (46.1)	109 (53.4)	5.57 (1.32)
Perc. ease of use 1	1 (0.5)	100 (48.1)	107 (51.4)	5.56 (1.21)
Perc. ease of use 2	7 (3.3)	113 (53.8)	90 (42.9)	5.21 (1.34)
Perc. ease of use 3	2 (1.0)	88 (43.3)	113 (55.7)	5.50 (1.26)
Perc. ease of use 4	18 (8.5)	111 (52.6)	82 (38.9)	4.92 (1.49)
Job relevance 1	0 (0)	80 (40.2)	119 (59.8)	5.86 (1.19)
Job relevance 2	3 (1.5)	66 (33.0)	131 (65.5)	5.98 (1.27)
Output quality 1	4 (2.0)	84 (42.0)	112 (56.0)	5.54 (1.28)
Output quality 2	8 (4.0)	95 (47.3)	98 (48.8)	5.25 (1.47)
Result demonstrability 1	4 (2.0)	81 (40.9)	113 (57.1)	5.61 (1.40)
Result demonstrability 2	3 (1.5)	73 (36.3)	125 (62.2)	5.76 (1.27)
Result demonstrability 3	2 (1.0)	88 (44.0)	110 (55.0)	5.66 (1.25)
Subjective norm 1	9 (4.3)	106 (51.0)	93 (44.7)	5.21 (1.49)
Subjective norm 2	5 (2.4)	118 (57.0)	84 (40.6)	5.15 (1.40)
Image 1	15 (7.5)	119 (59.8)	65 (32.7)	4.74 (1.62)
Image 2	29 (14.6)	106 (53.3)	64 (32.2)	4.45 (1.77)
Image 3	19 (9.5)	118 (59.0)	63 (31.5)	4.64 (1.65)

**Table 6:** Positive aspects of the application of computer technology.

Positive aspects of the application of computer technology	Access to information	“*My experience working with electronic registration is very positive, allows me quick access to the patients’ database. A computerized work environment has created an order for me at work.*”
	Efficiency and improvement in nursing practice	“*Since the introduction of a computerized sheet, I make the registration and reporting in a clear, immediate manner. There is access to a lot of data in a very short period, which in my opinion increases the efficiency of the work.*”
	Collaboration between nursing staff and computer staff	“*Some of the nursing staff had difficulty adapting to a computerized sheet, but thanks to the support of the nurse in charge, and the staff of the computer unit*, *the transition to electronic registration was not terrible overall.*”
	Prevention of errors in registration and reporting	“*A lot of information can be received in a short period, the writing is legible, and it prevents mistakes in registration. Today I do not doubt that it is easier for the entire nursing staff to function with electronic registration.*”

**Table 7:** Difficulties in applying computer technology.

Difficulties in applying computer technology	Lack of knowledge in using a new computer system	“*At first it was very difficult because we did not know the system.*”
	Difficulty adapting to a new registration and reporting method	“*I know that some of the nursing staff had difficulty adjusting to a computerized sheet.*”
	Time-consuming	“*At first there were many complaints about the lack of time for managing the treatment because most of the treatment time was spent on registering on the computer.*”
	A sense of increased technical engagement	“*In my opinion, the computerized sheet does create order, but it also creates technical work without the nurses’ need to think about how the recorded data will be concentrated and what it is used for; everything remains at a technical level of registration*”.

## Data Availability

The data supporting this study’s findings are available from the corresponding author upon reasonable request.

## Abbreviations and Acronyms

TAM	Technology Acceptance Model
EMS	Electronic Medical Systems
mHealth	Mobile Health
IT	Information Technology

## Appendix A. Interview Questions

1. Please tell me about yourself, how old are you, and what is your educational background?
2. How many years have you been a nurse, where do you work today, and what is your professional experience?
3. Is there a computerized registration and reporting system in the department in which you work, and what is your degree of use of this system?
4. Please tell me about an event or experience you had at the professional level, after the introduction of a computerized system.
5. How do you describe the role of the nurse in the technological age?
6. To what extent do you feel you belong to a functioning generation in the current technological age?
7. How do you describe your performance in the department following the use of EMS?
8. Do you have any suggestions regarding the implementation of the computerization process in the nurses’ job?
